# Innate and Adaptive Immune Responses Against Microsporidia Infection in Mammals

**DOI:** 10.3389/fmicb.2020.01468

**Published:** 2020-06-26

**Authors:** Yinze Han, Hailong Gao, Jinzhi Xu, Jian Luo, Bing Han, Jialing Bao, Guoqing Pan, Tian Li, Zeyang Zhou

**Affiliations:** ^1^State Key Laboratory of Silkworm Genome Biology, Southwest University, Chongqing, China; ^2^Chongqing Key Laboratory of Microsporidia Infection and Control, Southwest University, Chongqing, China; ^3^Department of Pathology, Albert Einstein College of Medicine, The Bronx, NY, United States; ^4^College of Life Sciences, Chongqing Normal University, Chongqing, China

**Keywords:** microsporidia, mammal host, immune response, innate immunity, adaptive immunity

## Abstract

Microsporidia are obligate intracellular and eukaryotic pathogens that can infect immunocompromised and immunocompetent mammals, including humans. Both innate and adaptive immune systems play important roles against microsporidian infection. The innate immune system can partially eliminate the infection by immune cells, such as gamma delta T cell, natural killer cells (NKs), macrophages and dendritic cells (DCs), and present the pathogens to lymphocytes. The innate immune cells can also prime and enhance the adaptive immune response via surface molecules and secreted cytokines. The adaptive immune system is critical to eliminate microsporidian infection by activating cytotoxic T lymphocyte (CTL) and humoral immune responses, and feedback regulation of the innate immune mechanism. In this review, we will discuss the cellular and molecular responses and functions of innate and adaptive immune systems against microsporidian infection.

## Introduction

Microsporidia are obligate intracellular parasites that infect nearly all vertebrates and invertebrates, including immunocompetent and immunocompromised humans. The Microsporidia phylum is composed of at least 200 genera and 1400 species (Cali et al., 2017). At least 17 species within nine genera (*Anncaliia, Encephalitozoon, Enterocytozoon, Microsporidium, Nosema, Pleistophora, Trachipleistophora, Tubulonosema, Vittaforma*) of microsporidia have been reported to be able to infect humans (Fayer and Santin-Duran, 2014; Han and Weiss, 2017). The human-infecting microsporidia are recognized as opportunistic pathogens, and frequently reported in immunocompetent and immunocompromised individuals, such as AIDS patients, cancer patients, transplant recipients, children and the elderly (Lores et al., 2002; Tumwine et al., 2002; Mor et al., 2009; Abu-Akkada et al., 2015; Wang et al., 2018; Ghoyounchi et al., 2019). Moreover, some microsporidia have a broad host range and can be transmitted among animals and humans, leading to zoonotic or interspecies transmission of microsporidiosis (Li et al., 2019; Udonsom et al., 2019).

When infecting, microsporidian spores extrude a polar tube, through which sporoplasms inside the spores are transported into host cells for development and proliferation (Franzen, 2005; Franzen et al., 2005b; Han et al., 2020). Inside host cell, the pathogen has to fight against host immune systems, including innate immunity and adaptive immunity. A few years ago, several reviews summarized studies in the early time on the immune responses of mammals against microsporidian infections (Khan et al., 2001; Ghosh and Weiss, 2012; Valencakova and Halanova, 2012). These very early analyses had partially indicated that both immunities are crucial to the resistance against microsporidian infections, and had found that immune cells including macrophages, dendritic cells (DCs) and CD8+ T cell, and cytokines like IL-12 and IFN- γ are activated and contribute to the immune defense (Mathews et al., 2009). In recent years, some new and further studies have revealed more cellular and molecular interactions between host immune system and microsporidia. In this review, we will systematically discuss the cellular and molecular responses to microsporidian infections from aspects of innate and adaptive immunities, respectively.

## Innate Immune Response to Microsporidian Infection

The innate immune system serves as the first line of defense and plays important roles in non-specific responses against infections. The innate immune system is composed of tissue barrier, innate immune cells, innate immune molecules, and cytokines (Beutler, 2004; Turvey and Broide, 2010; Sokol and Luster, 2015). The innate immune cells including macrophages, DCs, Natural Killer Cells (NKs) and innate-like lymphocytes have been proved to be essential for responses against microsporidian infections (Didier et al., 2010; Lawlor et al., 2010; Moretto et al., 2012). The innate immunity cannot completely clear microsporidian infection, but is necessary to activate the responses of adaptive immunities for a clearance.

### Macrophage Response to Microsporidian Infection

**FIGURE 1:**
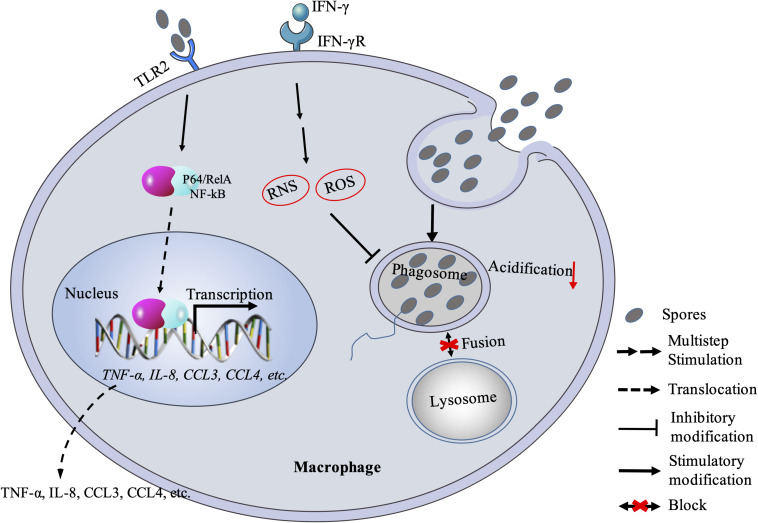
Macrophage response against microsporidian infection. Macrophage recognizes microsporidia via MyD88-dependent TLR2 and stimulates downstream expression of cytokines and chemokines. Macrophage can also kill the pathogen with RNS and ROS mechanism. Besides, microsporidia can block the fusion between phagosome and lysosome. RNS, reactive nitrogen species; ROS: reactive oxygen species.

Macrophages originate from blood monocytes, differentiate in tissues, and are involved in the detection, phagocytosis and destruction of harmful organisms. Macrophages can also present antigens to T cells and initiate inflammation by releasing cytokines to activate other immune cells. During microsporidian infection, macrophages can recognize the pathogens via pattern recognition receptors (PRRs) on the surface (Fischer et al., 2008a), and subsequently active defense mediators, such as chemokines, cytokines, reactive nitrogen, and radical oxygen, to control pathogen dissemination (Mathews et al., 2009). Macrophages are highly plasticity, can be activated and generate classically activated (M1) and alternatively activated (M2) types according to the activation state and functions, respectively (Martinez and Gordon, 2014). IFN-γ and lipopolysaccharide (LPS) are important activators for polarization of M1, which has intense phagocytic activity and higher microbicidal activity with NO and can secrete IL-12 and IFN-γ (Martinez and Gordon, 2014). It has been found that M1 can significantly reduce *Encephalitozoon* infection (Didier and Shadduck, 1994; Didier, 1995; Fischer et al., 2008b) depending on reactive nitrogen species (RNS) and reactive oxygen species (ROS) (Didier, 1995; Didier et al., 2010; Gonzalez-Machorro et al., 2019; Figure 1). The RNS- and ROS-deficient mice were shown to bear significantly higher peritoneal pathogen load and need longer time to eliminate the infection compared to the wild type of mice (Didier et al., 2010). Nonetheless, the deficient mice finally survived against *Encephalitozoon cuniculi* infection (Didier et al., 2010). Moreover, the phagocytosis of microsporidia significantly increased in LPS-activated murine macrophages, and the growth of pathogens inside the macrophages was inhibited, while the inhibitory effect lost after 72 h (Gonzalez-Machorro et al., 2019). These findings suggest that macrophages can control microsporidian infection to a certain extent, but the complete elimination likely requires other immunities. In addition, it was found that macrophage activities were associated with B-1 cells in microsporidian infection. In mice with B-1 cells, peritoneal macrophages had an M1 profile. In B-1 cell deficient mice, however, *E. cuniculi* modulated macrophages to M2, which is less phagocytic capacity and index and microbicidal activity, and inside which spore germination was observed, suggesting that B-1 cells are important in the modulation of macrophage in *E. cuniculi* infection (Pereira et al., 2019).

In fact, studies have tried to understand that how microsporidia escape the macrophage immunity. Early studies proved that microsporidian spores in vacuoles were able to block phagosome acidification and fusion with secondary lysosomes (Weidner and Sibley, 1985; Figure 1). Spore-containing phagocytic vacuoles fused with lysosomes can kill partial pathogens, while some sporoplasms can still escape from maturing lysosomes so that enter into host cytoplasm for successful infection (Franzen, 2005; Franzen et al., 2005b). When failing to inhibit the proliferation of microsporidia, macrophages can release chemokines (CCL3, CCL4) with TLR2-NF-κB signaling pathway to recruit phagocytes (Fischer et al., 2008a; Figure 1). However, this recruitment mechanism can be hijacked by the pathogens for infecting more monocytes and expanding the monocyte-derived-macrophage infections (Fischer et al., 2007). Meanwhile, the macrophage itself may also be hijacked as a “Trojan horse” to carry the parasites to other parts of the host body and spread the infection (Mathews et al., 2009). Furthermore, the macrophage immunity can be modulated by microsporidia via cytokines. In human macrophages infected with *Encephalitozoon*, the expression of IL-10 increased (Franzen et al., 2005a). This is consistent with the later study by Pereira et al. (2019) that macrophages were polarized to M2 type, in which the IL-10 expression is upregulated and the pathogens can escape from killing. Besides, the IL-10 belongs to an anti-inflammatory cytokine and can inhibit the production of nitric oxide and activity of macrophages and Th1 cells during infection (Popi et al., 2004; Couper et al., 2008). It was documented that early expression of IL-10 delayed the production of IFN-γ (Braunfuchsova et al., 1999), which was found to play an important role in macrophage-activating and resisting the microsporidian infection (Achbarou et al., 1996; El Fakhry et al., 1998, 2001; Khan and Moretto, 1999; Rodriguez-Tovar et al., 2016).

### DCs Response to Microsporidian Infection

Dendritic cells are essential antigen presentation cells and thus function as a bridge between innate and adaptive immune systems (Mellman and Steinman, 2001). Antigens presented by DCs to T cells promote the adaptive immunity via activating naïve lymphocytes into effector T cells, which will significantly boost immunity against the infection (Steinman and Hemmi, 2006; Bieber and Autenrieth, 2020). Besides, DCs can secrete many cytokines, such as IL-12 and IFNs, which will trigger adaptive immune responses against the foreigner invasions (Mellman and Steinman, 2001; Trinchieri, 2003; von Stebut and Tenzer, 2018).

**FIGURE 2:**
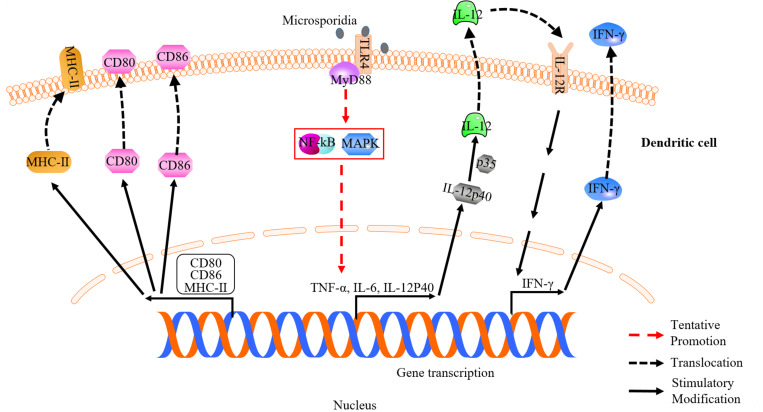
Dendritic cell (DC) response against microsporidian infection. Surface molecules of CD80, CD86, MHC-II and toll-like receptor 4 are up-regulated in infected DC. Upregulation of CD80, CD86, and MHC-II is essential for induction of optimal T-cell response. DC can recognize microsporidia via TLR4. IL-12 and IFN-γ secreted by DC help to optimize and prime effector T cell immune response, respectively.

It was reported that IFN-γ and IL-12 play vital roles in DCs response against microsporidian infection. The IFN-γ secreted by DCs is important for priming the gut intraepithelial lymphocytes response (IEL) against *E. cuniculi* infection. Murine DCs lacking IFN-γ failed to induce IEL response and led to ineffective suppression of the infection (Moretto et al., 2007, 2012). The IL-12 is also an important cytokine responding to microsporidian infection. The mutant mice lacking IL-12 was found to be susceptible to the infection (Khan and Moretto, 1999), and CD8+ T cells showed poor immune response (Moretto et al., 2010). In infected DCs, IL-12 production was strongly induced, while the p40^–/–^ DCs lacking IL-12 failed to develop robust CD8+ T cell-mediated immune response to *E. cuniculi* infection (Moretto et al., 2010). Studies also showed that the IL-12 can be induced during early infection of DC by *E. cuniculi*, suggesting that IL-12 is important to the initiation of innate immunity (Lawlor et al., 2010; Figure 2). Moreover, the PRRs of DCs, like Toll-like receptors (TLRs), are crucial for pathogen-recognition. It was shown that TLR4 is a key factor for DCs response (Figure 2), and is also essential for the expression of costimulatory molecules (CD80, CD86, and MHC class II), which induce optimal antigen-specific CD8+ T cells response to *E. cuniculi* infection (Lawlor et al., 2010). In *Enterocytozoon bieneusi* infection, however, TLR4 is not required for DCs response, but the myeloid differentiation factor 88 (MyD88) involved in Toll-like signaling pathway is needed (Zhang et al., 2011). Therefore, DCs probably recognize different microsporidia via different TLRs.

**FIGURE 3:**
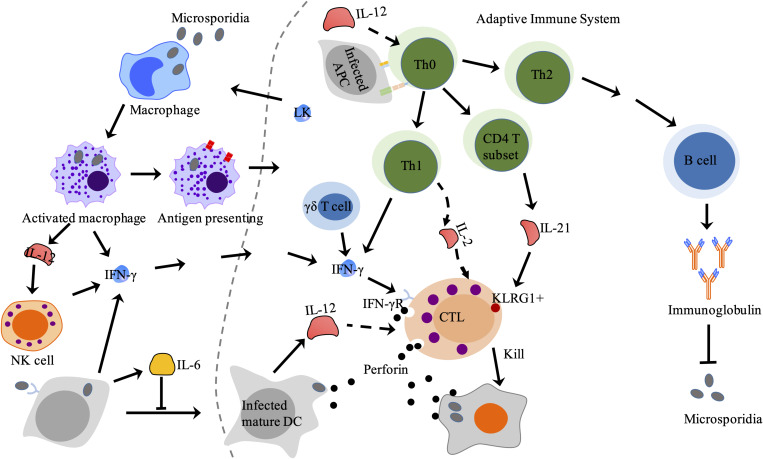
Adaptive immune response against microsporidia in mammals. CTL is activated by APC and cytokines from innate immune cells and Th cell and then lyses target cell via perforin-dependent cytotoxicity. Antibodies produced by effector B lymphocytes can partially block microsporidian infection. LK produced by lymphocytes stimulates macrophage to secret IFN-γ. LK, lymphokines; CTL, cytotoxic T lymphocytes; Th cell, helper T cell; APC, antigen presenting cell.

On the other hand, the differentiation of DCs was found to be inhibited by microsporidia via an IL-6-dependent mechanism so that the parasites can escape from stronger immune defense (Bernal et al., 2016; Figure 3). In addition, the ability of priming antigen-specific T cell response in aged host decreases at the microsporidia-infected gut mucosal site (Moretto et al., 2008; Gigley and Khan, 2011). The maturation of DCs can be inhibited by the programmed death-ligand 1 (PD-L1), which expression is increased in aged mice leading to decrease of mature DCs and T cells immunity (Gigley and Khan, 2011). This probably is a reason why the aged is susceptible to microsporidian infection (Lores et al., 2002).

### NKs Response to Microsporidian Infection

The NKs are a type of cytotoxic lymphocytes and critical to the innate immune system. NKs can not only lyse infected cells, but also secret cytokines like IFN-γ to induce immunity of antigen specific T cells (Yokoyama, 2005). It was found that the activity of NKs was enhanced in mice infected with *E. cuniculi* (Niederkorn et al., 1983), and the number of NKs increased upon early infection of *E. cuniculi* but decreased to a normal level in late infection stage (Khan et al., 1999). Further studies indicated that NKs can also produce IFN-γ after stimulated by IL-12 secreted by macrophages (Braunfuchsova et al., 1999; Salat et al., 2004; Figure 3).

### Intraepithelial Lymphocytes Response to Microsporidian Infection

The intestinal mucosa is an important defense line against microsporidia by inducing strong intraepithelial lymphocytes (IELs) response (Moretto et al., 2004, 2007, 2012). The population of IELs accompanied with the secretion of cytokines, such as IFN-γ and IL-10, rapidly increases in early infection by *E. cuniculi*. And the activated IELs are cytotoxic to infected syngeneic macrophages, which can kill more than 60% antigen-specific target cells (Moretto et al., 2004). These findings indicated that the early expansion of IELs not only serves as the first defense line, but also offers immunoregulators. Besides, the IFN-γ produced by DCs from mucosal sites is crucial for evoking antigen-specific IEL responses against microsporidia in the small intestine (Moretto et al., 2007).

### Apoptosis Response to Microsporidian Infection

Apoptosis is a form of programmed cell death that also responses to pathogen infection and plays significant roles in the control of the immune response (Knodler and Finlay, 2001; Luder et al., 2001; Higes et al., 2013).

**FIGURE 4:**
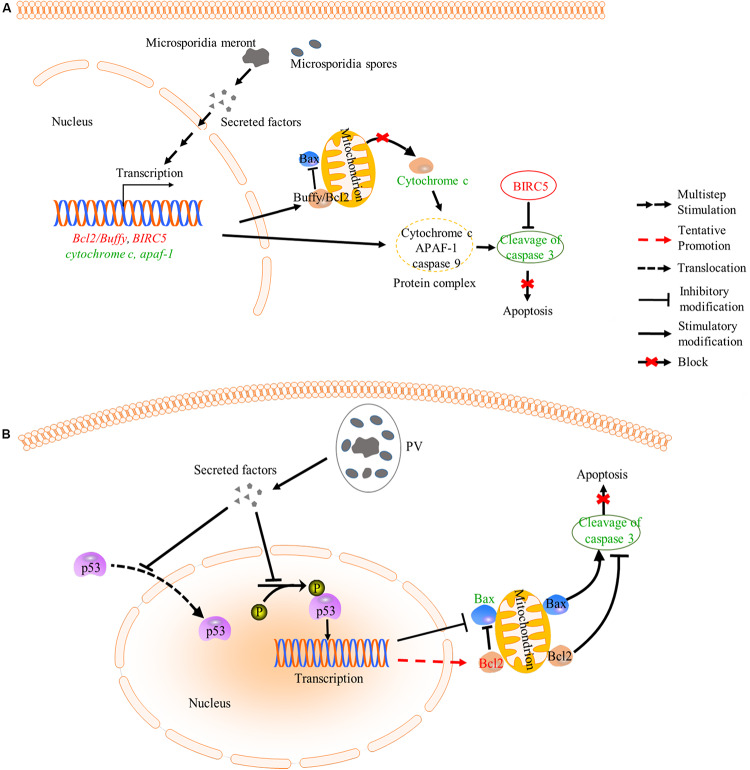
Inhibition of apoptosis by microsporidia. **(A)** Microsporidian species which directly expose to host cytoplasm can inhibit the release of cytochrome c (CYT-C) by up-regulating the expression of *Bcl2*/*Buffy*. Besides, down-regulated expression of *cyt-c* and *apaf-1* may cause reduction of cytochrome-APAF1-Caspase 9 complexes, which decrease cleavage activity of caspase 3. And BIRC5 belongs to a member of IAP which can inhibit the capase3 activation. These changes caused the inhibition of host apoptosis. **(B)** Microsporidia species which live inside PV can inhibit host apoptosis by decreasing the phosphorylation and nuclear translocation of p53, which also leads to suppression of cleavage activity of caspase 3. words in red: up-regulated gene; Words in green: down-regulated gene; PV, parasitophorous vacuole.

Intracellular parasites like microsporidia and *Toxoplasma gondii* can suppress host apoptosis in order to have more time for proliferation (Nash et al., 1998; Heussler et al., 2001; Higes et al., 2013; He et al., 2015; Kurze et al., 2015; Sinpoo et al., 2017; Sokolova et al., 2019). Microsporidia can inhibit host apoptosis via modulating apoptosis-related proteins. Buffy and BIRC5 are two host factors that suppress apoptosis. The buffy is a Bcl-2 like protein and can inhibit caspase-dependent cell death by activating downstream gene expressions in *Drosophila* (Quinn et al., 2003). And the BIRC5 is a member of the inhibitors of apoptosis family (IAP), which can inhibit caspase activation to reduce apoptosis (Heussler et al., 2001; Martin-Hernandez et al., 2017). Microsporidian *Nosema ceranae* and *Nosema apis* were reported to suppress host apoptosis via up-regulating expression of the *buffy* and *BIRC5* (Higes et al., 2013; Martin-Hernandez et al., 2017; Figure 4A). In mitochondria-mediated apoptosis pathway, the tumor suppressor protein p53 is activated for recruiting Bax (pro-apoptosis protein member of Bcl-2 family) to mitochondrial membrane. Then the Bax promotes the release of cytochrome c (CYT-C) from mitochondria to cytoplasm, where the CYT-C binds to apoptosis protease activated factor1 (APAF1). Finally, the APAF1, cytochrome c and caspase-9 assemble and form a protein complex to facilitate cell death (Heussler et al., 2001; Figure 4A). Microsporidian *Nosema bombycis* can also inhibit host apoptosis by down-regulating the expression of *apaf1* and *cyt-c* and up-regulating the expression of *buffy*, so that reduce caspase-3 activity and inhibit host apoptosis (He et al., 2015). Besides, *Encephalitozoon* infection can suppress apoptosis of Vero cells by inhibiting the cleavage of caspase-3, phosphorylation and translocation of p53 (del Aguila et al., 2006; Figure 4B). In the apoptosis process, the p53 activated the expressions of *p21* and *Bax* to induce apoptosis (Miyashita et al., 1994). However, expressional changes of the *Bcl-2* and *Bax* are not observed in *Encephalitozoon* infected Vero cells for unknown reasons (del Aguila et al., 2006). A previous study showed that *T. gondii* prevented the activation and cytosol-mitochondrial targeting of Bax but not the protein levels to inhibit host apoptosis (Hippe et al., 2009). It is possible that *Encephalitozoon* also utilize mechanisms similar to that of the *T. gondii* to modulate host Bax.

In a recent study, the expressions of 84 apoptosis-related genes were investigated in *E. cuniculi* and *Vittaforma corneae* infected THP1 cell line. As a result, both pathogens can manipulate intrinsic apoptosis pathway (Sokolova et al., 2019). The assayed pro-apoptosis genes including *LTA, CARD8, TRADD, BAX, CASP3, CASP1, CASP4, CASP9, BP2, DARK1*, and *BCLAF1* are all down-regulated, while some anti-apoptosis genes, such as *ABL1, BAG1, BAG3, BCL2, TNFRSF1A*, and *CD40LG*, are all up-regulated upon infection. This study suggests that host apoptosis pathway is deeply modulated by the pathogen. Similar to *Encephalitozoon*, other intracellular parasites, such as *T. gondii, Trypanosoma cruzi*, and *Cryptosporidium parvum*, were also found to inhibit host apoptosis via similar mechanisms (Heussler et al., 2001; Luder et al., 2001; Mammari et al., 2019), indicating that intracellular pathogens can modulate host apoptosis pathway with similar or conserved strategies.

### Antimicrobial Peptides Response to Microsporidian Infection

Antimicrobial peptides are important components of the host innate immune system. It was reported that lactoferrin (Lf), lysozyme (Lz), human beta defensin 2 (HBD2), human alpha defensin 5 (HD5), and human alpha defensin 1 (HNP1) are capable of inhibiting microsporidian spore germination and reducing enterocytes infection (Leitch and Ceballos, 2008). For example, the germination of *Encephalitozoon hellem* spore can be inhibited by HNP1. And the germination of *Anncaliia algerae* spore can also be blocked by Lf, HBD2, HD5, and HNP1 (Leitch and Ceballos, 2008). However, this inhibitory ability of antimicrobial peptides is not applicable to all microsporidian species. The germination of *Encephalitozoon intestinalis* spores cannot be inhibited by any of the peptides listed above (Leitch and Ceballos, 2008). It is likely that microsporidia have different germination mechanisms.

## Adaptive Immune Response Against Microsporidian Infection

Adaptive immunity is broader and more efficient protection against non-self antigens than innate immune system. Adaptive immunity involves T lymphocytes-mediated cellular immunity and B lymphocytes-mediated humoral immunity, which have a tightly regulated interplay with antigen-presenting cells and facilitate the pathogen-specific elimination (Bonilla and Oettgen, 2010). When the innate immunity fails to block pathogen invasion, the adaptive immunity with production of antibodies and cytotoxic T lymphocytes (CTLs) will be triggered to resolve the infection. And T cell-mediated immunity plays a principal role in protection against microsporidia lethal infection (Valencakova and Halanova, 2012).

### Humoral Immune Response to Microsporidian Infection

Humoral immunity is a very effective way to scavenge pathogens from the host (Casadevall, 2018). Treatment of Vero E6 cells with anti-exospore monoclonal antibody P5/H1 could reduce *E. cuniculi* infection *in vitro.* One explanation for the inhibition is that P5/H1 neutralizes sensitive epitopes on *E. cuniculi* spores so that the growth of the pathogens was suppressed (Schmidt and Shadduck, 1984). Antibodies binding to spores led to macrophages making more efficient phagocytosis and increase oxidative burst (Sak et al., 2004). In addition, the P5/H1 could prolong the survival of microsporidia-infected SCID mice, which were reconstituted with CD4+ T lymphocytes (Sak et al., 2006). Further studies showed that the protective effect of the antibodies on SCID mice depends on IFN-γ (Salat et al., 2008).

Microsporidia like *E. cuniculi* are able to induce a strong antibody response. Increase of IgA, IgM, and IgG were detected in infected mice (Cox, 1977; Sak and Ditrich, 2005; Omalu et al., 2007; Figure 3). However, these antibodies cannot protect IFN-γ knockout mice and athymic BALB/c (nu/nu) from death upon *E. cuniculi* infection (Schmidt and Shadduck, 1983; Salat et al., 2004; Valencakova and Halanova, 2012), suggesting that the antibody alone is not powerful enough to completely clear the infection.

### T Cell-Mediated Immune Response to Microsporidian Infection

T cell-mediated immune response in host is crucial for prevention of infection. CD4+ and CD8+ T cells were found induced in infected mice and rabbits (El Naas et al., 1999; Soto-Dominguez et al., 2020), and were important in resisting against microsporidia (de Moura et al., 2019). And among T cell population, the CD8+ T cell subtype plays a major role during infection (Moretto et al., 2004). However, the activation of CD8+ T cell is associated with other immune cells and cytokines (Moretto et al., 2010; Langanke Dos Santos et al., 2017). The CD4+ T cells can be activated by infection and will develop to effective T cells. The number of CD4+ T cells increased upon microsporidian infection in mice (El Naas et al., 1999). In addition, spleen cells from wild mice inoculated with *E. intestinalis* were shown to have elevated levels of IFN-γ and IL-2 (El Fakhry et al., 2001), which are cytokines secreted by Th1 and involved in activation of CD8+ T cells. Adoptive transfer of pure CD4+ T cells can prolong the survival of SCID mice (Salat et al., 2006). However, adoptive transfer of CD4+ T cells from IFN-γ deficient mice cannot prolong the survival of SCID mice (Salat et al., 2008). These results indicate that CD8+ T lymphocytes-independent protection against the infection can be mediated by CD4+ T lymphocytes and the protective immunity is mediated by IFN-γ, which is a potential activator of macrophages. In other studies, the co-inhibitory receptor killer-cell lectin like receptor G1 (KLRG1) was found to be expressed in NKs and antigen-experienced T cells (Henson and Akbar, 2009). During the response against *E. cuniculi*, the KLRG1+ T cell subset was the majority of polyfunctional effector CD8+ T cells (Bhadra et al., 2014). A more recent study demonstrated that IL-21 secreted by the CD4+ T cells was important for inducing KLRG1^+^ effector CD8+ T cells against microsporidian infection (Moretto and Khan, 2016; Figure 3). These studies indicate that the CD4+ T cells play roles against microsporidian infection. However, mice lacking CD4+ T cell still can survive from microsporidian infection (Moretto et al., 2000), suggesting that CD4+ T cell is not a key immune defense against the infection.

Strong CD8+ T cell responses were also observed during microsporidian infection (Khan et al., 1999). The CD8^–/–^ mice became more susceptible to *E. cuniculi* infection (Moretto et al., 2000). And SCID mice, deficient in T and B cells, reconstituted with CD8+ T cell-contained splenocytes resolved the infection (Braunfuchsova et al., 2001; Salat et al., 2002). These results suggest that the CD8+ T lymphocytes play a crucial role in resisting against *E. cuniculi*. And this protection depends on the activity of CD8+ CTLs, which lead to lysis of infected cells by perforin pathway (Khan et al., 1999; Figure 3). Moreover, trigger of CD8+ T cells into CTLs had been documented. The activation of the CD8+ T cells can be evoked by CD4+ T cells. For example, mice lacking CD4+ T cells show a lower CTLs response to virus infection (Matloubian et al., 1994). However, CD4+ T cell deficiency does not affect the effector CD8+ T cell responding against *E. cuniculi* infection (Moretto et al., 2000), suggesting CD4+ T cell is not the sole activator of CD8+ T cell response and another reason is associated with the route of infection (Moretto et al., 2004; de Moura et al., 2019). It was found that other immune cell types like DC, γδ T cell, and B-1 cell are also key roles in modulating the antigen-specific CD8+ T cell immunity (Moretto et al., 2001; Langanke Dos Santos et al., 2017). The γδ T cells were reported to be important for establishing primary immune response against pathogen infection by producing cytokines (Berguer and Ferrick, 1995; Ferrick et al., 1995; Figure 3). It was found that the γδ T cell significantly increases in early infection of mice by *E. cuniculi*, while lacking of γδ T cell causes down-regulation of CD8+ cell immune response (Moretto et al., 2001). In addition to γδ T cell, the B-1 cell was found to be able to decrease host susceptibility to *E. cuniculi* (da Costa et al., 2016). In microsporidia-infected BABL/c XID mice, which are B-1 cell deficient, the population of CD8+ T cell decreases compared with that of infected BABL/c mice (Langanke Dos Santos et al., 2017). This suggests that the B-1 cell can increase the immunity of the CD8+ T lymphocytes, in addition to increasing pro-inflammatory cytokines and modulating M1 prolife (Pereira et al., 2019). Probably, B-1 cell behaves as an APC to increase population of CD8+ T cell and promotes the activation of CD4+ T cell (Hardy, 2006; Margry et al., 2013). Besides, the CD8+ T cell response can also be activated by DCs-secreted IL-12 during infection by *E. cuniculi* (Moretto et al., 2010; Figure 3). These findings demonstrate that CD8+ T cell is vital in responses against microsporidia and also explain why CD8+ T cell can be induced in the absence of the CD4+ T cell.

In general, T cell-mediated immune protection is essential for elimination of microsporidian infection. However, microsporidian spores still can remain in some organs of immunocompetent mice and may become a source of infection onset (Kotkova et al., 2013; Sak et al., 2017). This raises an important question that how microsporidia escape the strong immunities.

## Conclusion

The innate and adaptive immune systems play vital roles against microsporidian infection. Further understanding of host immune responses against the infection will greatly help with the diagnosis and treatment of microsporidiosis. The innate immune system not only directly resists the infection but also triggers the adaptive immunity. Innate immune cells, such as macrophages, DCs, γδ T cell, and NKs, can control microsporidia to some extent, and also secret cytokines and chemokines to assist both the innate and adaptive immunities against the infection. The cytokine IL-12 and IFN-γ can help to clear the parasite by activating related immune responses. However, early expression of IL-10 is benefit for the pathogen growth by negatively regulating the IFN-gamma expression. Yet the relation between IL-10 and microsporidia remains to be clarified. Besides, it is worth noting that lymphokines can also activate macrophages to kill microsporidia (Schmidt and Shadduck, 1984; Figure 3). With the innate immunities, however, the host cell cannot completely clear microsporidia probably due to the evasive mechanism of the pathogens (Weidner, 1975; Bernal et al., 2016).

To completely clear the infection, the host needs to initiate the adaptive immunity. First of all, the CTL can be activated to restrict the proliferation of microsporidia by lysing infected cells via perforin pathway (Figure 3). In addition, increase of IgA, IgM, and IgG was observed in microsporidia-infected mice (Cox, 1977; Sak and Ditrich, 2005; Omalu et al., 2007). Furthermore, specific antibodies have been found to have strong inhibitory effects against microsporidia (Sak et al., 2004, 2006). In the adaptive immunity, antibodies play important roles in resisting against microsporidia. However, humoral responses to microsporidian infection have not been clearly illuminated and further studies with microsporidia-infected mice models are needed.

In summary, the interactions between microsporidia and host immune systems have been further studied, but are far to be fully elucidated. For example, what are the key host cells and factors that confer the defense effects. And what are the mechanisms that microsporidia modulate host immune responses. Hopefully, genomics and proteomics studies on microsporidia have provided some important clues and candidates to dissect the questions (Katinka et al., 2001; Mittleider et al., 2002; Akiyoshi et al., 2009; Corradi et al., 2010; Reinke et al., 2017).

## Author Contributions

TL and ZZ contributed to conception and design of the study. TL and YH wrote the first draft of the manuscript. HG, JX, JL, BH, JB, and GP wrote sections of the manuscript. All authors contributed to manuscript revision, read and approved the submitted version.

## Conflict of Interest

The authors declare that the research was conducted in the absence of any commercial or financial relationships that could be construed as a potential conflict of interest. The reviewer LW declared a shared affiliation with the author BH at the time of review.
